# Supplementary material for: Immunogenicity and Safety of Half-Dose Heterologous mRNA-1273 Booster Vaccination for Adults Primed with the CoronaVac® and ChAdOx1-S Vaccines for SARS-CoV-2
Source: Vaccines (Basel). 2024 Mar 22;12(4):344. doi: 10.3390/vaccines12040344 (PMC11053985; doi:10.3390/vaccines12040344)
Supplement: Supplementary file 1 [file vaccines-12-00344-s001.zip › vaccines-2896082-supplementary.pdf]

Supplementary 1. Gating strategy for intracellular cytokine staining (ICS).

Gating Strategy

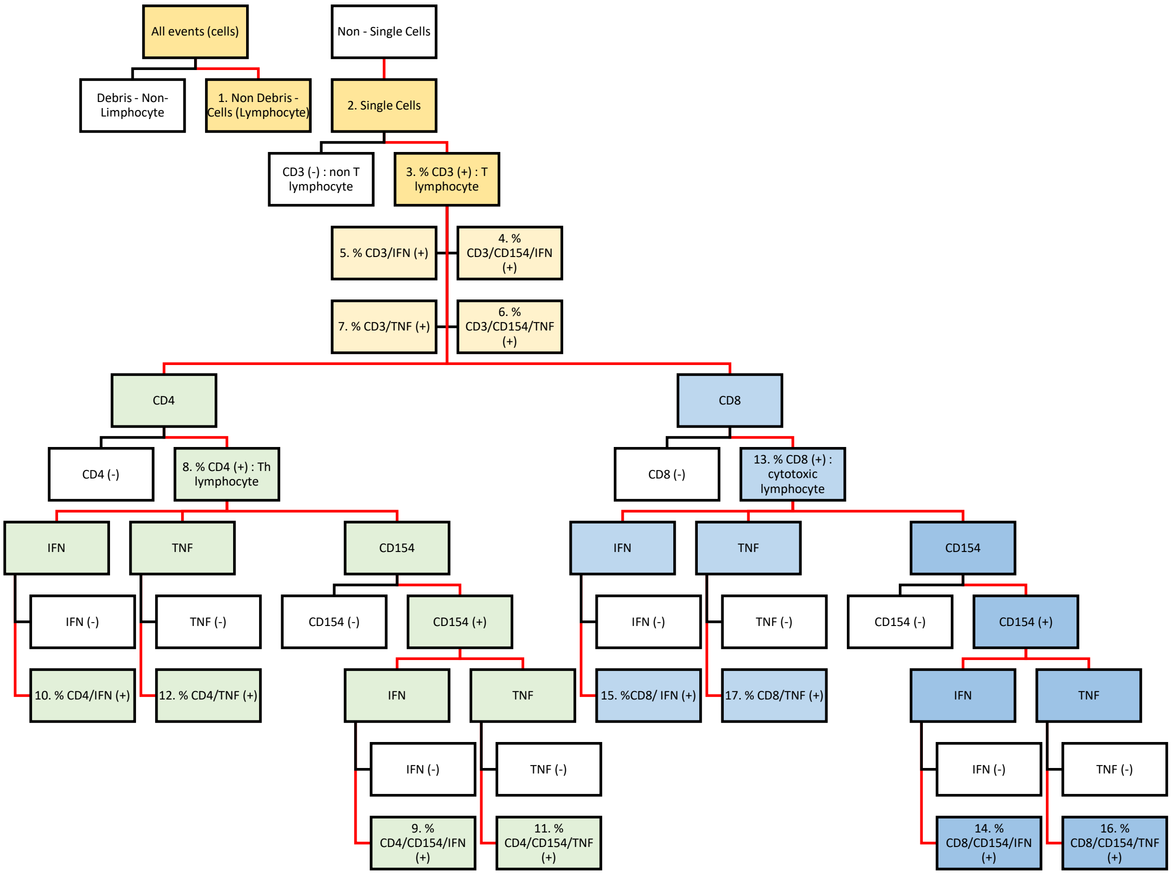

Output in percentage :

1. Non Debris – Cell (Lymphocytes)
2. Single Cells
3. % CD3 (+) & Viable % T Lymphocytes
4. % CD3+/CD154+/IFN+ (Q2)
5. % CD3+/IFN+
6. % CD3+/CD154+/TNF+ (Q6)
7. % CD3+/TNF+
8. % CD3+/CD4+
9. % CD3+/CD4+/CD154+/IFN+ (Q2)
10. CD3+/CD4+/IFN+
11. CD3+/CD4+/CD154+/TNF+ (Q6)
12. CD3+/CD4+/TNF+

13. % CD3+/CD8+
14. % CD3+/CD8+/CD154+/IFN+
15. % CD3+/CD8+/IFN+
16. % CD3+/CD8+/CD154+/TNF+
17. % CD3+/CD8+/TNF+

1	Non Debris – Cell (Lymphocytes)	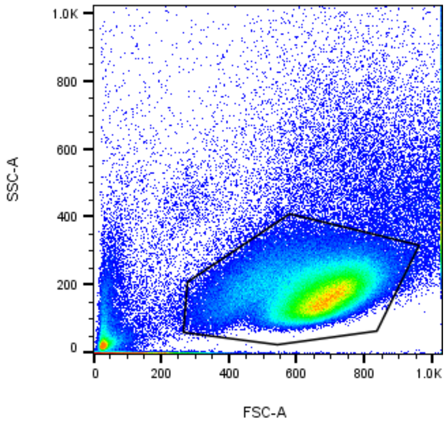<br>01-09-2022_AIM P06.fcs<br>Ungated<br>306477
2	Single Cells	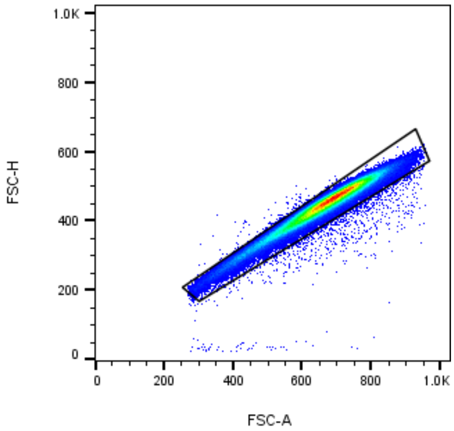<br>01-09-2022_AIM P06.fcs<br>Lymphocytes<br>216353

3.	% CD3 (+) & Viable<br><br>Marker :<br>CD3 : APC<br>Viable : Pacific Blue	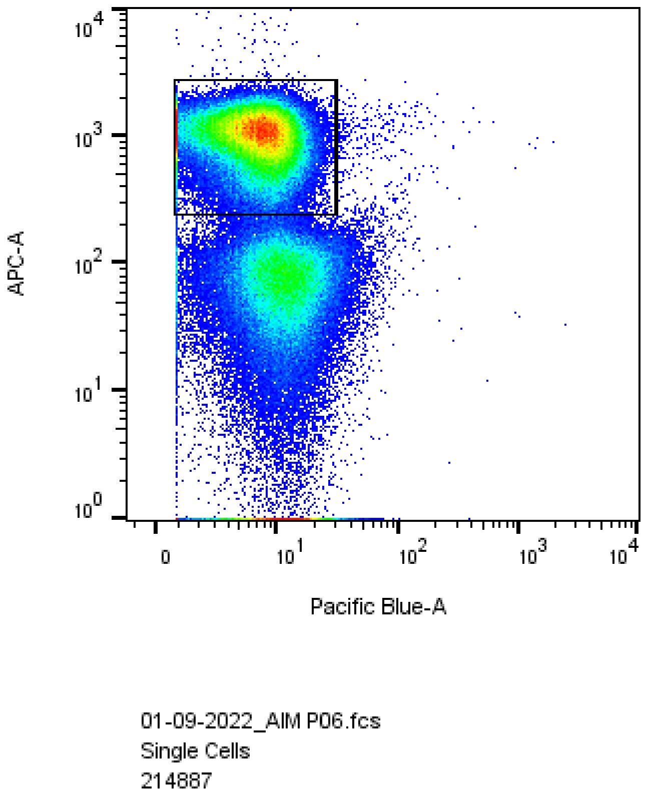
4	% CD3+/CD154+/IFN+ (Q2)<br><br>Marker :<br>CD154 : APC Cy.7<br>IFN gamma : PE<br><br>Graphic type :<br>Quadrant dot plot	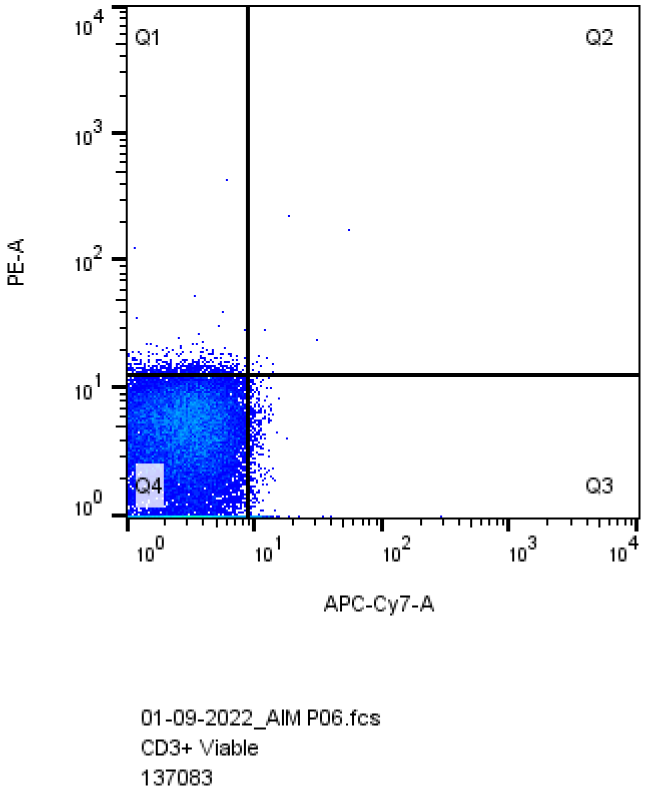

5	% CD3+/IFN+<br><br>Marker :<br>IFN Gamma : PE<br><br>Graphic type :<br>Histogram	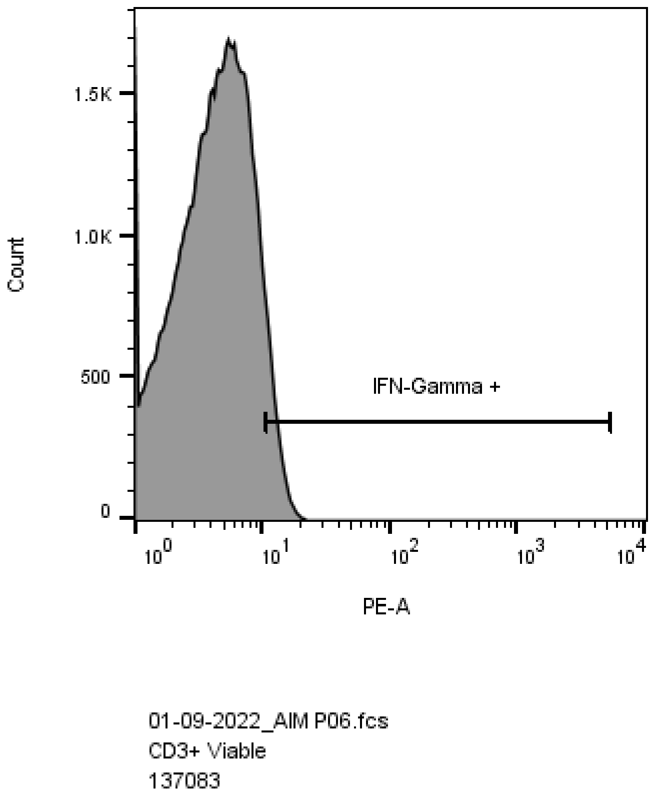
6.	% CD3+/CD154+/TNF+ (Q6)<br><br>Marker :<br>TNF : PE Cy.7	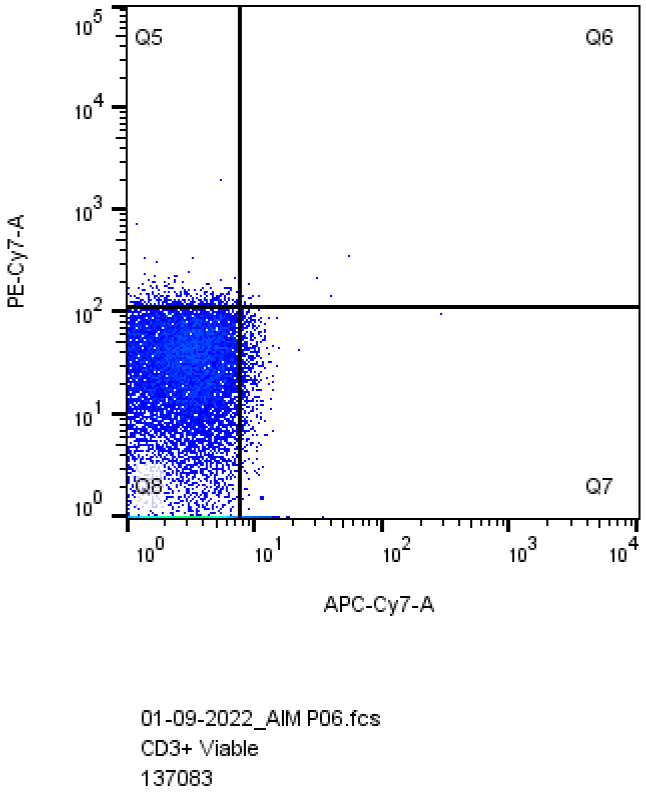

<table>
<tr><td>7.</td><td>% CD3+/TNF+</td><td>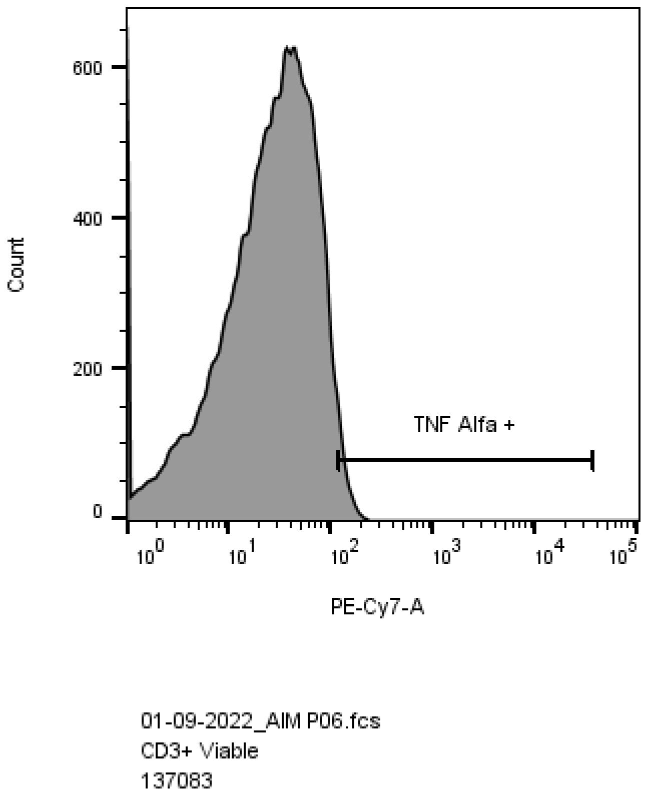
</td></tr>
<tr><td>8.</td><td>% CD3+/CD4+

Marker :
CD4 : FITC</td><td>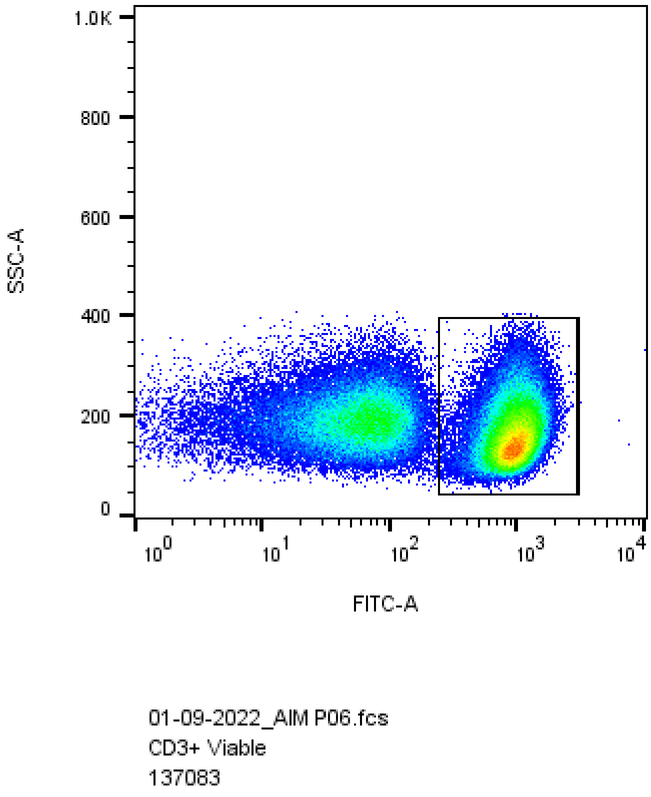
</td></tr>
</table>

<table>
<tr><td>9.</td><td>% CD3+/CD4+/CD154+/IFN+ (Q2)</td><td>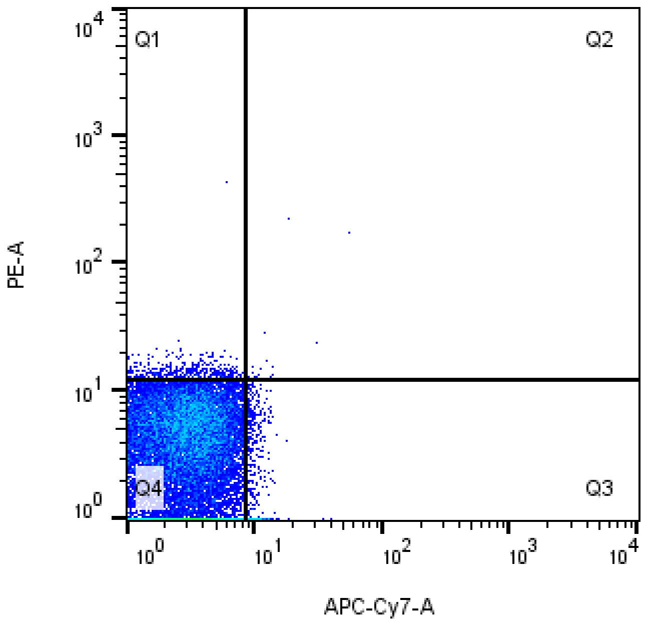

01-09-2022_AIM P06.fcs
CD4+
75158</td></tr>
<tr><td></td><td></td><td></td></tr>
<tr><td>10</td><td>CD3+/CD4+/IFN+</td><td>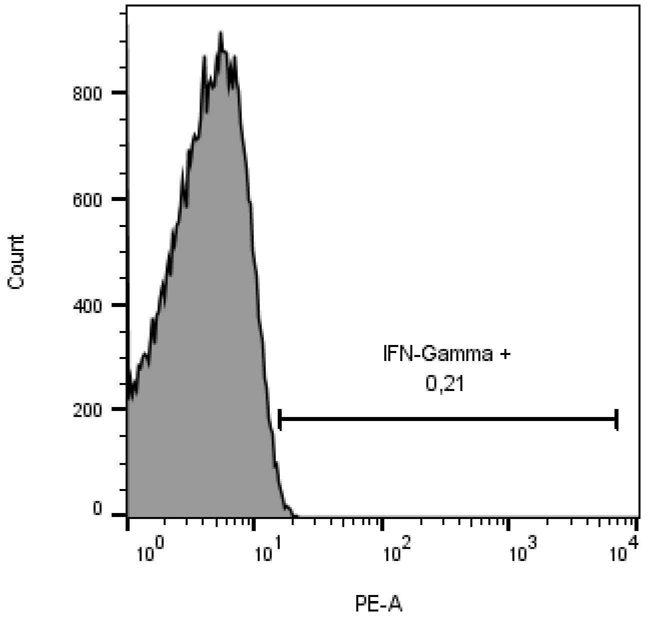

01-09-2022_AIM P06.fcs
CD4+
75158</td></tr>
</table>

11	CD3+/CD4+/CD154+/TNF+ (Q6)	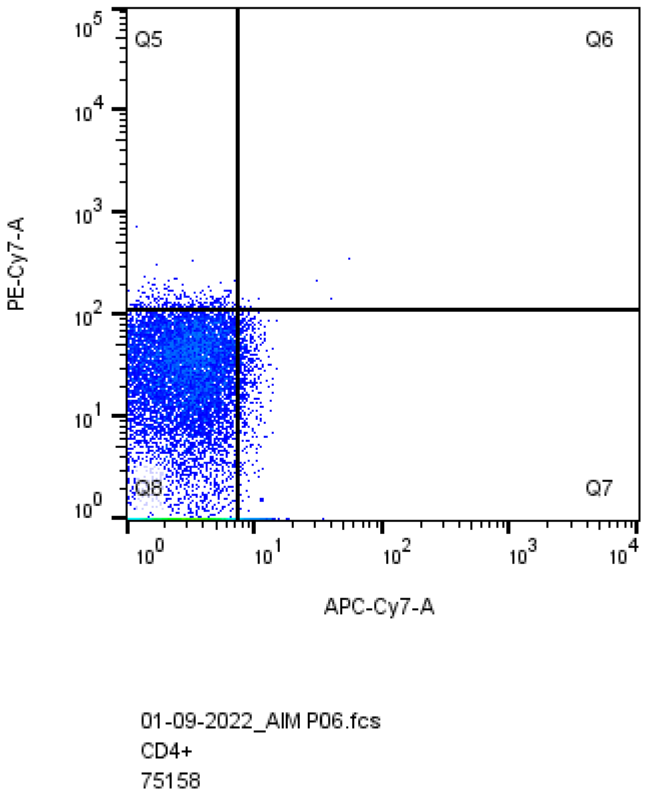
12	CD3+/CD4+/TNF+	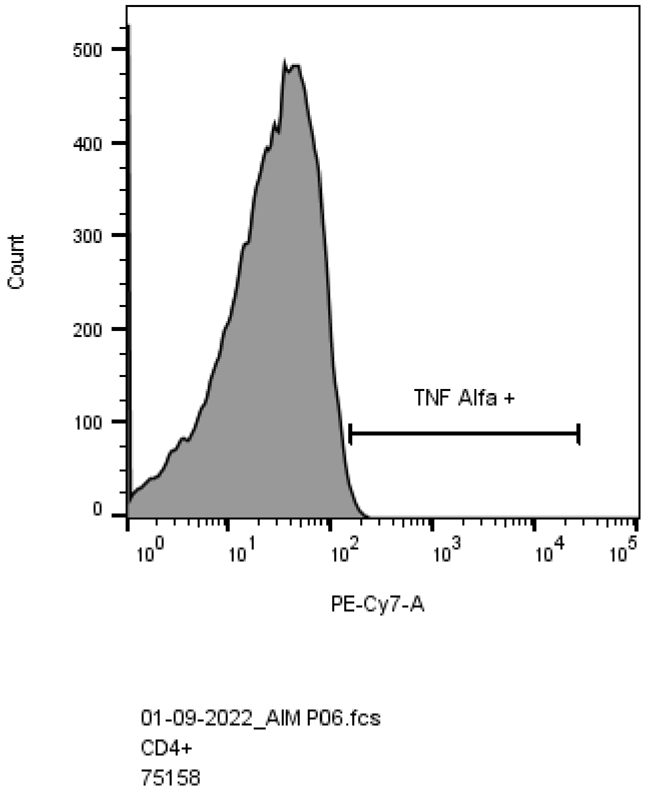

13	% CD3+/CD8+<br><br>Marker :<br>CD8 : Amcyan	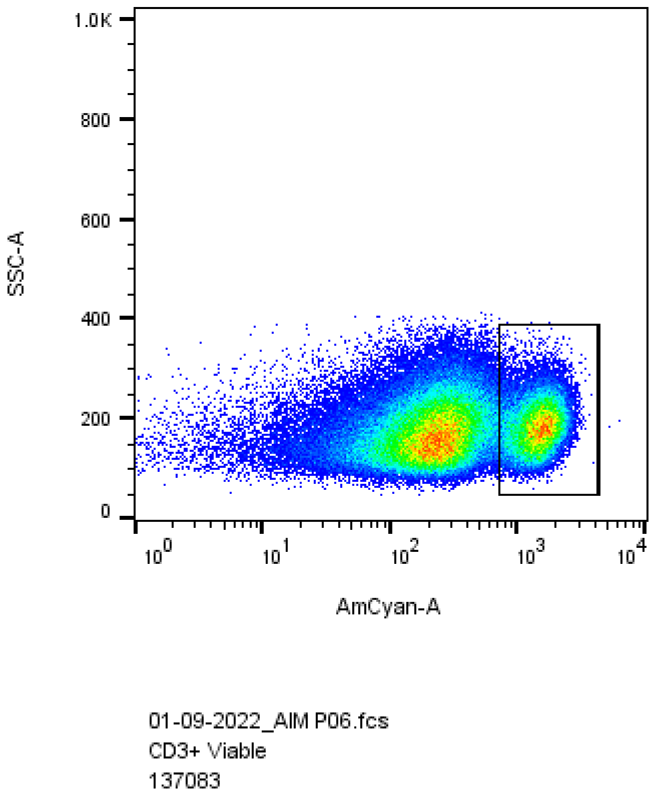
14	% CD3+/CD8+/CD154+/IFN+	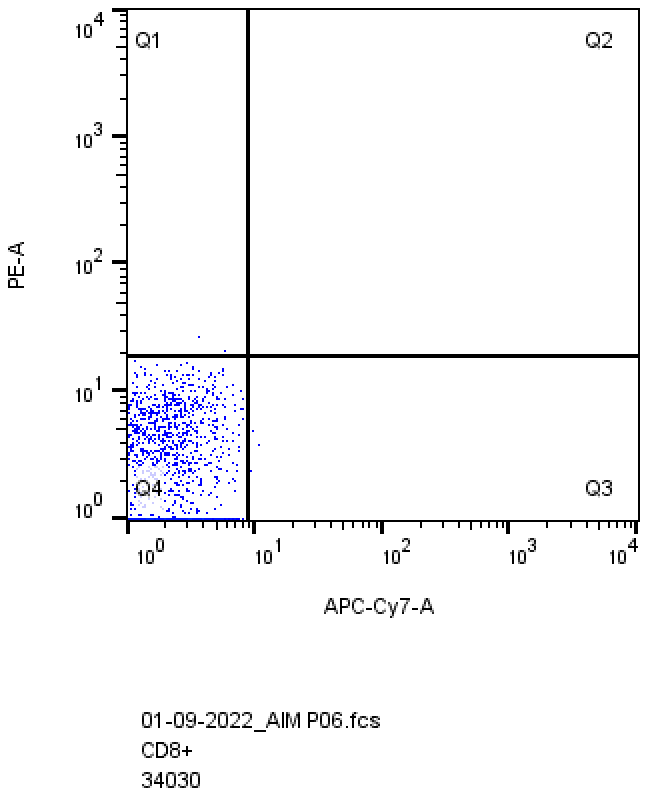

15	% CD3+/CD8+/IFN+	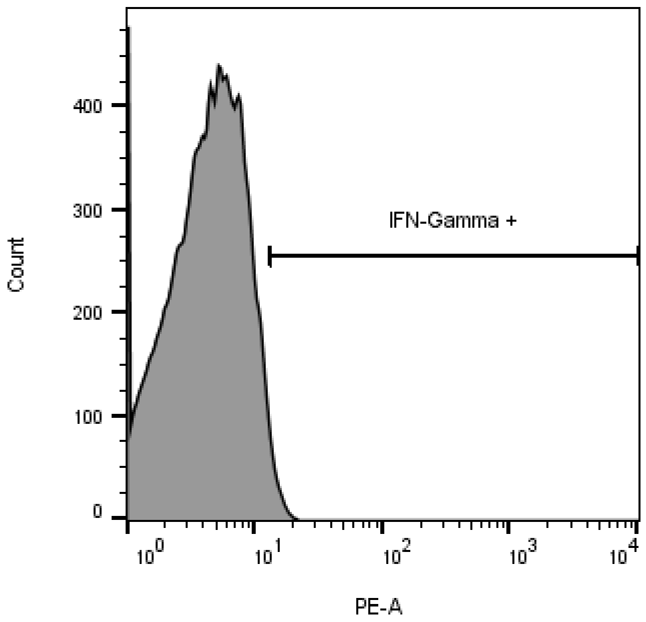<br>01-09-2022_AIM P06.fcs<br>CD8+<br>34030
16	% CD3+/CD8+/CD154+/TNF+	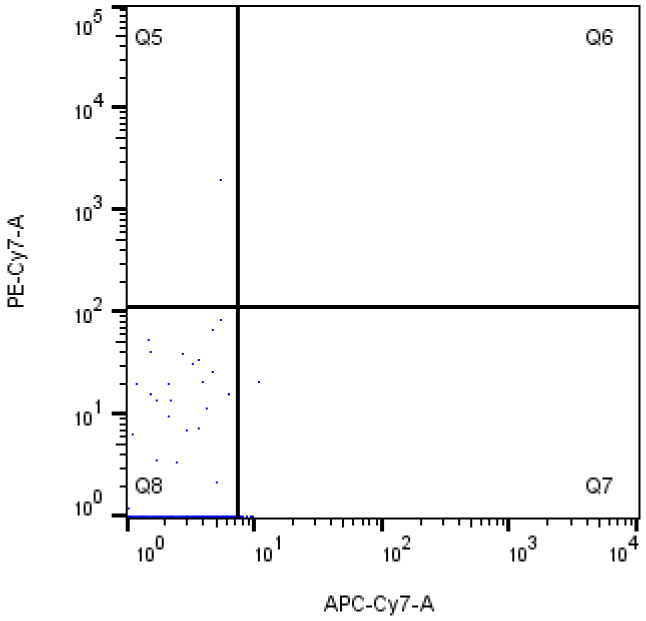<br>01-09-2022_AIM P06.fcs<br>CD8+<br>34030

17	% CD3+/CD8+/TNF+	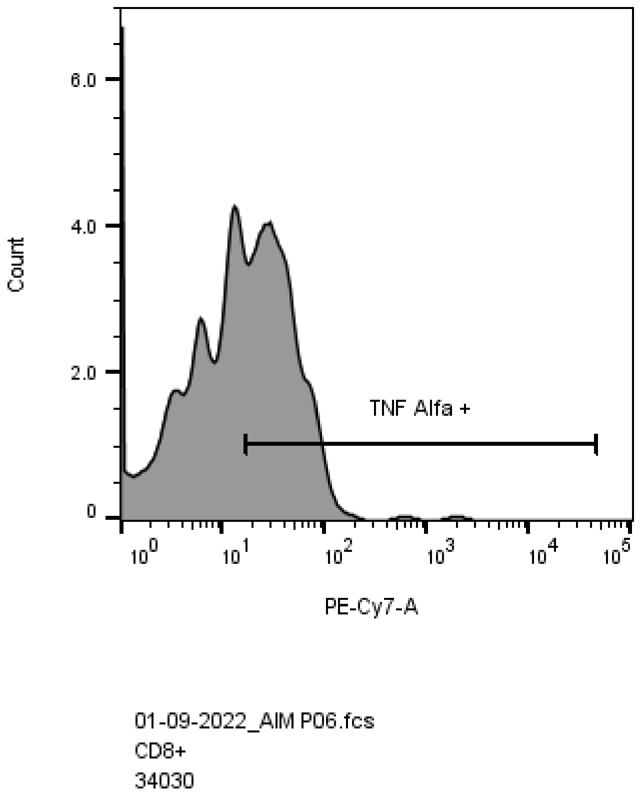
